# A FPGA Embedded Web Server for Remote Monitoring and Control of Smart Sensors Networks

**DOI:** 10.3390/s140100416

**Published:** 2013-12-27

**Authors:** Eduardo Magdaleno, Manuel Rodríguez, Fernando Pérez, David Hernández, Enrique García

**Affiliations:** 1 Department of Fundamental and Experimental Electronic, Physics and Systems, Universidad de La Laguna, Avd. Francisco Sanchez s/n, 38203 La Laguna, Spain; E-Mails: mrvalido@ull.es (M.R.); dhernane@ull.es (D.H.); enrique.jose.garcia.gonzalez@gmail.com (E.G.); 2 Department of Statistics, Operations Research and Computation, Universidad de La Laguna, Avd. Francisco Sanchez s/n, 38203 La Laguna, Spain; E-Mail: fdoperez@ull.es

**Keywords:** embedded web server, FPGA, remote monitoring, NIOS, TTP/A

## Abstract

This article describes the implementation of a web server using an embedded Altera NIOS II IP core, a general purpose and configurable RISC processor which is embedded in a Cyclone FPGA. The processor uses the μCLinux operating system to support a Boa web server of dynamic pages using Common Gateway Interface (CGI). The FPGA is configured to act like the master node of a network, and also to control and monitor a network of smart sensors or instruments. In order to develop a totally functional system, the FPGA also includes an implementation of the time-triggered protocol (TTP/A). Thus, the implemented master node has two interfaces, the webserver that acts as an Internet interface and the other to control the network. This protocol is widely used to connecting smart sensors and actuators and microsystems in embedded real-time systems in different application domains, e.g., industrial, automotive, domotic, *etc.*, although this protocol can be easily replaced by any other because of the inherent characteristics of the FPGA-based technology.

## 1. Introduction

Nowadays, due to the Internet and embedded webservers, it is possible to carry out technological remote monitoring operations at a very low cost [1]. In fact, embedded web servers have a growing presence in a wide range of areas related to the commercial electronics and industrial applications [2,3]. These systems are characterized by a device dedicated to monitoring microsystem networks in real time or to perform any given task automatically without requiring human intervention [4]. Usually, most of these devices are implemented using PCs or microcontrollers, however, FPGAs are a viable alternative in the implementation of these systems since they add new features to traditional architectures based on microprocessors or microcontrollers. For example, the FPGA technology makes the embedded webserver small-sized (portable), flexible, reconfigurable and reprogrammable with the advantages of good customization, cost-effectiveness, integration, accessibility and expandability [5]. We can design the hardware, software and core simultaneously, which greatly reduces the design cycle [6]. FPGA technology also offers extremely high-performance signal processing. All these features allow us to implement in a single device an embedded webserver that is executed using a soft or hard microcontroller inside the FPGA chip [7]. This microcontroller can interact with IP cores or VHDL modules that perform specific processing hardware and other tasks.

**Figure 1.**
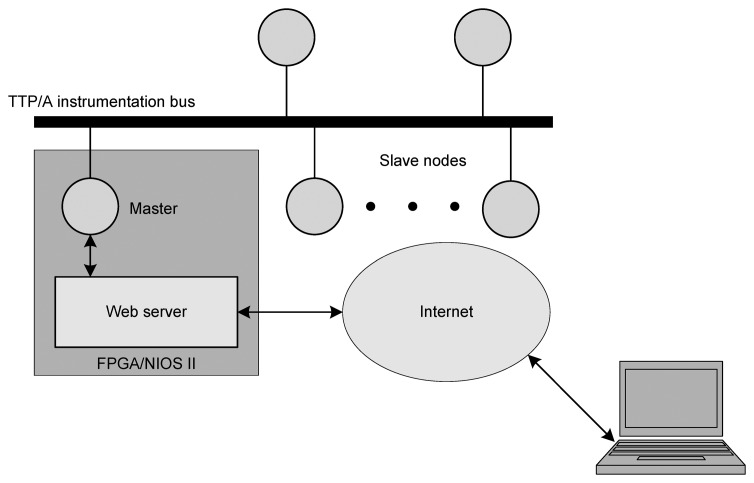
The implemented system general scheme.

The aim of this work is to implement an embedded web server in an FPGA to control and monitor a network of instruments or smart sensors. The web server acts as an interface between the Internet and the master node that controls the sensor network (Figure 1). Also, the implemented embedded webserver is able to control any sensor or instrument network simply by changing the driver between the server and the master node. In our case, as an application example, we used the webserver to control a network of smart sensors based on the Time-Triggered Architecture, Class A (TTP/A) protocol, a low speed and low cost version of TTP [8].

This example application based on TTP/A encapsulates and hides the technical details from the physical transducers and provides a concise abstract interface of its features. We are actually working in a project which main objective is to develop a standard interface for integrating smart sensors or micro-electromechanical system (MEMS or microsystem) based on a hierarchical communications system governed by a master node and we can obtain a standardized interface using TTP/A [9,10]. Fundamentally, we have selected this protocol for the implementation of the system by this fact.

We have developed an embedded processor in FPGA. It is able to communicate with all nodes of the sensor network through the TTP/A standard interface. It also interfaces the network with the user through a webserver.

The advantage of implementing the master node and the Internet interface in a FPGA system-on-chip, in comparison with a microcontroller system, is the implementation of a customizable architecture with an embedded webserver. This architecture is very flexible. We can connect different master peripheral modules that are developed in VHDL. These modules are modified according to the protocol that uses the smart sensor network. In this sense, we could have a universal embedded webserver using a VHDL library of existing smart sensor network protocols. The system configuration is very simple, all that is necessary to change is the VHDL module or compatible IP core of the network protocol.

Under this framework and in order to reach these goals, we have implemented the webserver using an Altera board and a Nios II embedded IP core, a configurable general purpose embedded RISC processor with embedded peripheral architecture, with the μClinux operating system [11–14]. We used a Boa server on this soft-architecture [15], a fast and light weight web server with CGI support. We also have implemented specific software including TTP/A master node to realize communication tasks. Also, we have implemented a software slave node in a conventional PC to check the whole system. The main contribution of this paper is the implementation an easy and flexible webserver interface to control and monitoring any smart sensor network or instrument just changing the protocol communication.

This work is structured in five sections including this. First, we describe the technology that has provided support for web server deployment. The following section shows the FPGA-based configuration that was used to implement a functional prototype with two aspects: driver protocol TTP/A and web server. The fourth section details the results and discussions. Finally, it ends with conclusions and future improvements.

## 2. System Description

This section presents the base technology used for hardware system development, the description of the smart sensor protocol (TTP/A) and the configuration of the Base System on FPGA.

### 2.1. NIOS II Soft Core Processor

**Figure 2.**
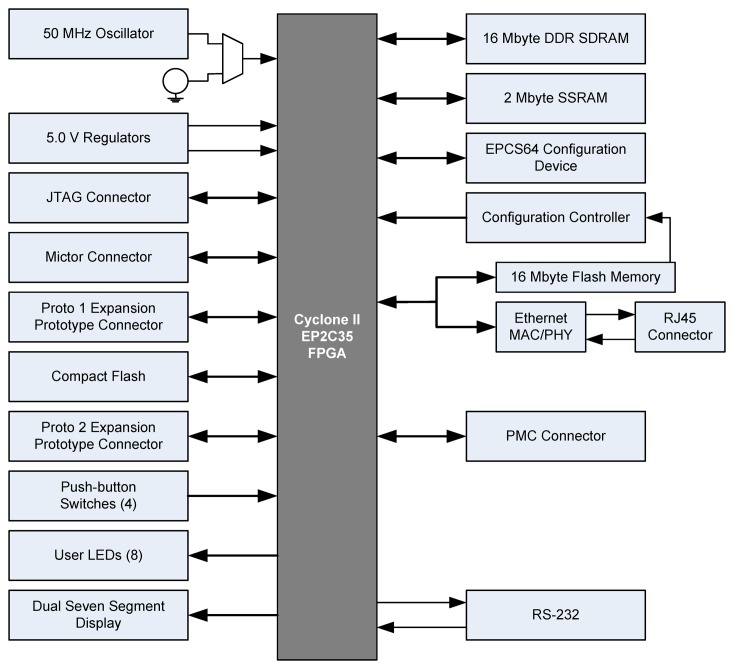
Development board scheme.

To implement the embedded web server, we have used the Altera Cyclone II EP2C35 [16]. This FPGA contains 33,216 logic elements, 105 memory blocks of 4 Kbits, 35 multipliers, 4 PLLs, 475-pin input/output and can operate at a maximum frequency of 260 MHz. It is located in the Nios II Embedded Development Kit. The development board scheme is depicted in Figure 2.

Traditionally, this kind of systems has been built using a general purpose processor implemented as Application Specific Integrated Circuits (ASIC) with a fixed architecture. A NIOS II Soft Core Processor is a microprocessor fully described in software, usually in HDL, which can be synthesized in FPGAs. A soft-core processor targeting FPGAs is flexible because its parameters can be changed at any time by reprogramming the device.

The Nios II microcontroller is an Altera development. It is a second generation 32-bit RISC-based architecture. The core of the system is scalable, being able to incorporate, for example customized instructions in arithmetic logic unit, and also peripherals to carry out specific functions and release the CPU of expensive work that would otherwise make the microcontroller slower in critical tasks.

The NIOS II family consists of three cores: fast (Nios II/f), economy (Nios II/e) and standard (Nios II/s). The Nios II/s core has been used in this prototype because it is the most common core. Furthermore, satisfactory results have been achieved using this core, which uses less hardware resources than Nios II/f.

### 2.2. Architecture and Network Protocol TTP/A

**Figure 3.**
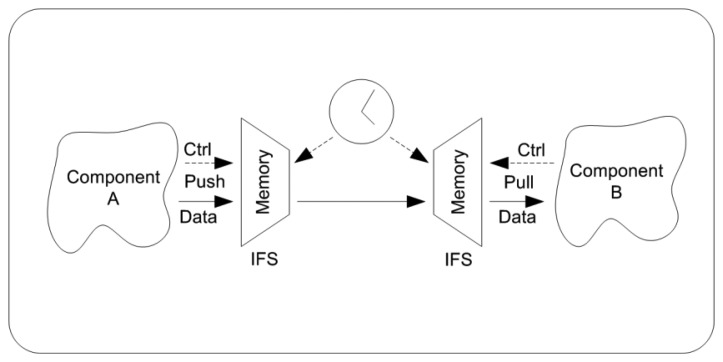
Decoupled flow control.

The communication between two network elements is based on a time-triggered communication where the data flow is controlled by a global clock. Each component has a memory that acts as source and destination for all data using pull and push styles respectively (Figure 3) [8]. Conceptually, this memory acts as an interface file system (IFS). This allows the data to be sent can be written in the memory through a push-type interface. The data transmission is overseen by a time-triggered communications model. After the transmission, the data sink reads the data through a pull-type interface. Values are stored in memory and can be interpreted as status messages until their contents are updated and overwritten. Possible conflicts between simultaneous memory read-write operations are avoided by managing the memory with a time-triggered protocol. One feature in this type of architecture is that all the nodes know the transmission formats and have access to the same system clock, thus every component knows the instant in which the protocol updates the memory. This smart transducer interface using IFS has the advantage of encapsulating and isolating internal complexity of slave nodes.

**Figure 4.**
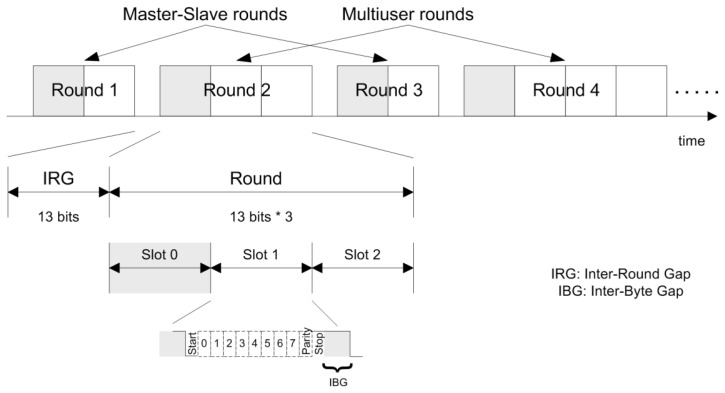
Format communication protocol TTP/A.

The interface protocol is controlled by an active master that supplies the synchronization to all the slave nodes. The communication is round-based. Every round starts with a fireworks byte sent by the master that is used for synchronization and round identification (Figure 4). Bus access conflicts are avoided by a strict time-division multiple access (TDMA) schedule for each round. A round consists of several slots and a slot is a unit for transmission of one byte of data. Each round is described in a round descriptor list (RODL) stored in every node into the IFS. This file contains the information about the actions performed by a node for a particular round (write, read, execute or idle) in each slot into a particular round. RODL files can be modified using special master-slave rounds.

The master node IFS also includes the ROSE (Round Sequence) file, where rounds are held to be executed. The controller of slave nodes is implemented in VHDL. The system can be resized according to the number of nodes in the network taking advantages of the VHDL configurability [9].

### 2.3. Configuring the Base System on FPGA

**Figure 5.**
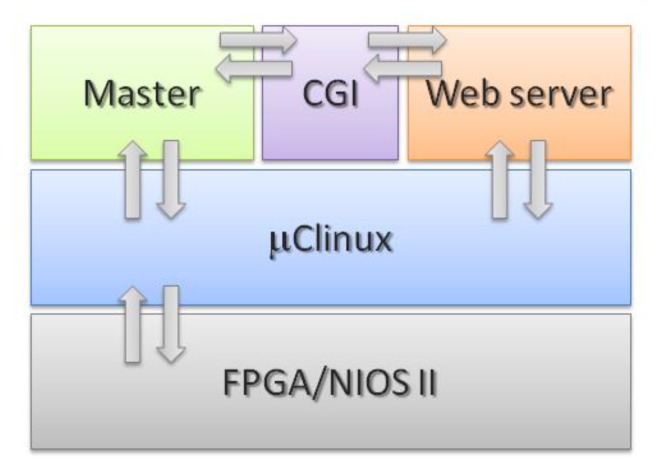
Architecture of the implemented system.

The architecture of the implemented system is represented in Figure 5. We have used a NIOS II FPGA as hardware technology. The operating system handles communication with hardware and deployed applications, *i.e.*, TTP/A master node controller and web server. The TTP/A master node controller and a web interface runs on a dynamic page web server using Common Gateway Interface (CGI). This has allowed a development consisting of layers, where each layer uses its lower layer to support processing and provides services to its upper layer.

First, the microcontroller is embedded in the FPGA NIOS II with the necessary interfaces and memory. This is implemented using the Altera Quartus II environment where it is added to NIOS II RS-232 interface for communication with the network nodes, RJ-45 interface for connecting the development board to an Ethernet network and communicate it globally and a JTAG interface for downloading information to the board to program it.

After setting up the microcontroller, we installed an open source system called μCLinux [13] with a Linux kernel version adapted to microcontrollers without MMU. This operating system has proved suitable for the realization of webservers in embedded systems and besides this, μcLinux has support and software applications [14].

**Figure 6.**
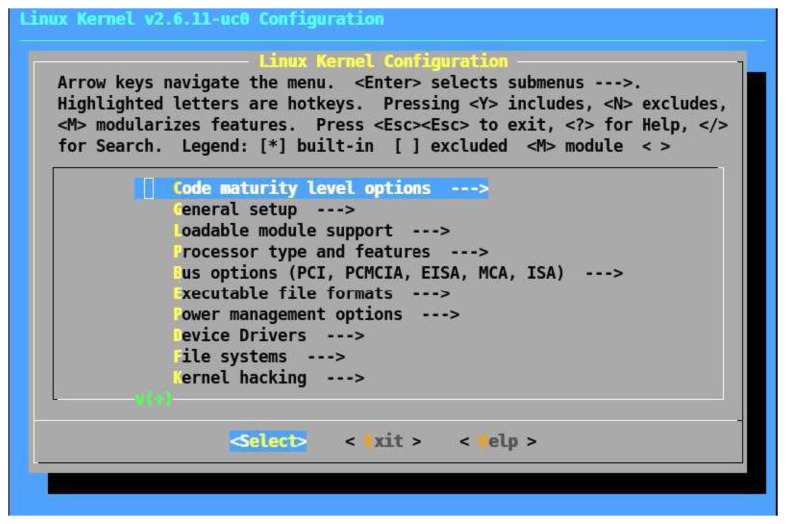
Kernel configuration menu.

The system is compiled from its sources so it requires a C cross compiler for NIOS II target machines running on x86 systems. Therefore, the system has been previously compiled on a workstation. The installation consists of the following steps:

- Configure NIOS II memory and ports mapping.
- Configure the kernel by the configuration menu (Figure 6): selection of the Cyclone II development board, Ethernet support, TCP/IP protocol, serial port, UNIX 98 pseudo-terminals (to add a Telnet server) and a serial console (only to install and configure the system)
- Compiling the kernel
- Download the compiled kernel to the board through SOPC Builder Development Tools, using NIOS II Integrated Development Environment (IDE)

**Figure 7.**
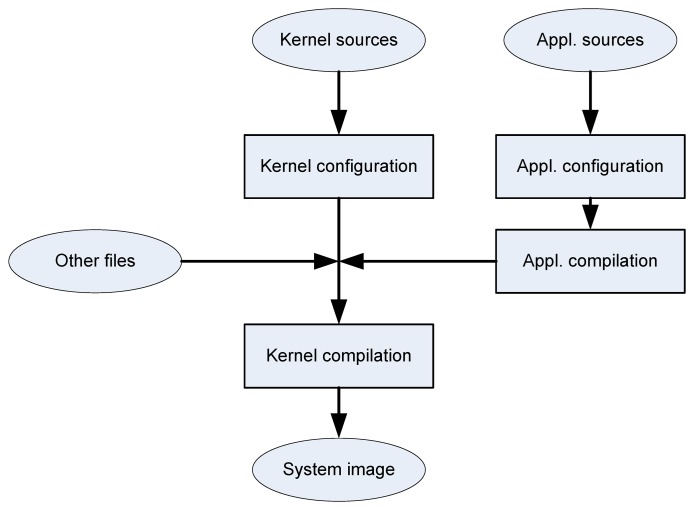
Obtaining the system image.

In Figure 7 is depicted a block diagram of the process to obtain images of the system, including the kernel and file system applications and settings. Based on the sources of the applications, executable files are created after setting up some parameters if necessary. It also generates files required to configure system boot and users.

Since it is necessary to include these files in the file system, we will copy them in the *rootfs* directory. This directory contains the system image to be generated and, later, loaded into the development board. The kernel is configured and finally compiled; as a result it creates a process from which the system image is built. In addition to the base configuration, the system has been supplemented with the installation of the following applications:

- Boa web server. This is a fast and light weight web server with CGI support [15].
- FTP server. To upload files and programs used in the board without having to continuously repeat the steps of compiling and loading the kernel, we installed an FTP server. Similarly to the web site, it was supplied by Microtronix.
- Telnet server. The telnet server was included to connect to the board regardless of the serial port, which will be busy performing communications tasks with slave nodes on the sensors network.
- Internet super-server (inetd). To launch the FTP and telnet servers according to the requests through the corresponding ports inetd was installed, supplied by Microtronix like other applications used.
- Users. To protect the system during the debugging process an user has been added to the system with restricted permissions.
- Startup Settings (inittab). In order to have the services available from the system boot, we have included relevant lines in the inittab file, which defines the behavior of the *init* process of system boot.

## 3. Implementing the Sensors Network Master Node

**Figure 8.**
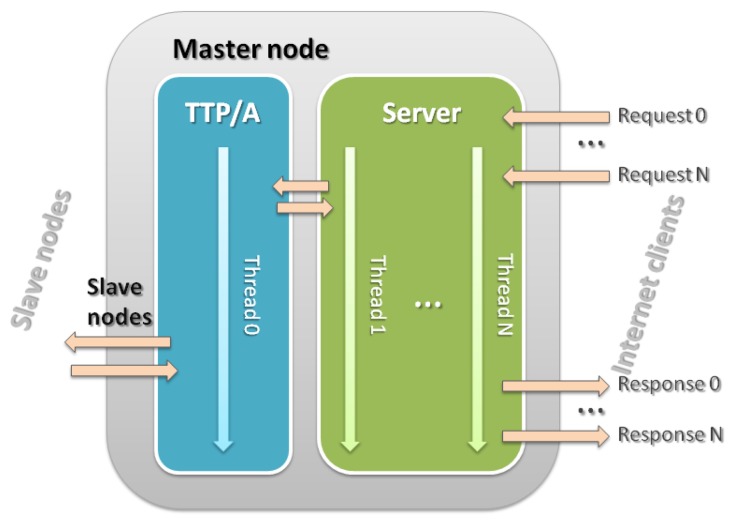
Architecture of the master node.

In our design we use the RS-232 interface existing in the development board to communicate with network nodes. The implemented master node is responsible for managing communication through this channel, to generate synchronization events and other tasks concerning itself and described in the previous section. The implementation was made using the C programming language, taking advantage of its power and the facilities it provides, such as system calls, *sockets* and *threads* [17]. Thus, the master may be seen as a running program that executes the part of the protocol TTP/A that corresponds to high priority. It also can be seen as a server that responds to external world requests for configuration and management (Figure 8). These requests are served through a low-priority thread and the use of *sockets* for remote access.

### 3.1. TTP/A Task

The execution of code directly related to the TTP/A protocol on the master node is done by dedicated *threads*. Since the TTP/A protocol dictates time requirements that must be fulfilled for the proper functioning of the system, a high priority execution is assigned to the thread. In this way we obtained a clear preference over other generated threads.

The main purpose of this thread is to execute all operations directly related to slaves, *i.e.*, operations that require sending and/or receiving information to/or from the sensors network, such as MSA/MSD rounds execution, multiuser rounds, RODL configuration and read/write memory.

Although the main and most time consuming task of the master is the bus arbitration by indicating notification of multiuser rounds execution, also master/slave rounds are inserted in order to complete other tasks. To achieve this goal, we implement a buffer capable of storing different sets of tasks which are executed in an orderly manner according to their arrival time.

The tasks that make use of this feature are those that are not part of multiuser rounds execution, and therefore do not require the same priorities of time. These tasks are:

- Slave programming: the master extracts from the task buffer all slaves programming information and transmits it to the concerned slave. In this case, transmitted data are raw data instead of rounds.
- Query memory: the master extracts from the task buffer the slave and file to request. After that, it makes requests through the RS-232 bus and stores the response; at the end of the request, it reports the conclusion of the task.

These high-level tasks are translated into bus-level TTP/A operations by MSA/MSD rounds interspersed with the execution of multiuser rounds.

**Figure 9.**
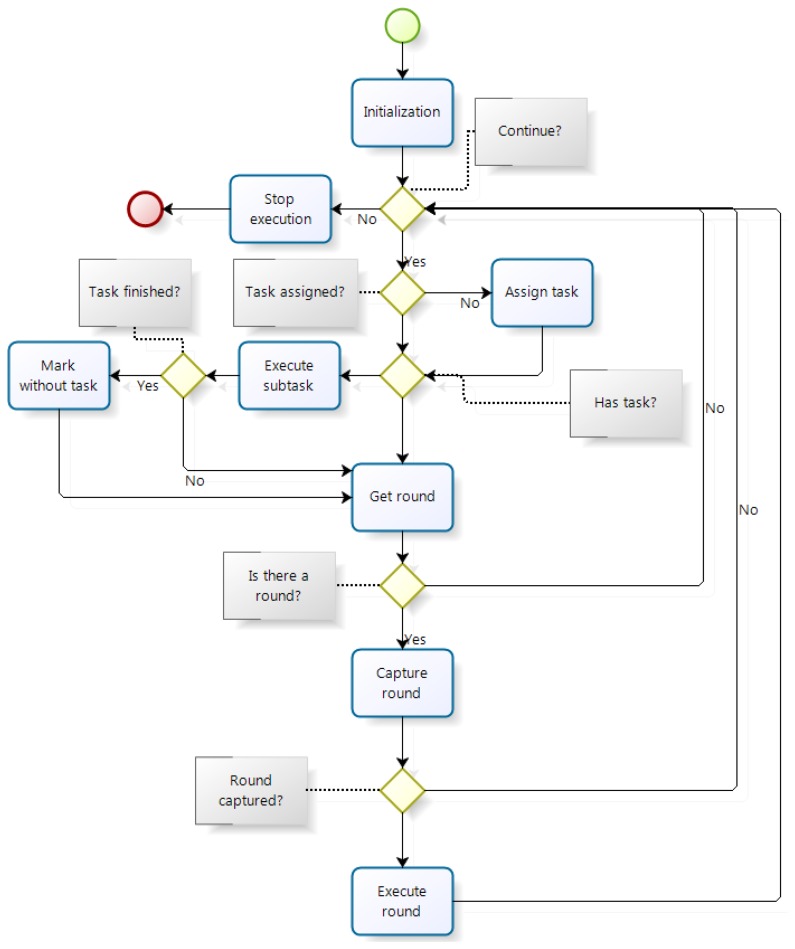
Flow of execution of the master thread.

The master execution flow, in its TTP/A aspect, is drawn in Figure 9. At the beginning of the thread the device boot occurs resetting all data to prepare for execution. Once the boot is completed the main execution loop is executed, which is completed unless it is aborted. In the case of an abort, the thread finishes execution and the program exits. If execution continues and enters the main loop, it checks if there is any task assigned and in case of not having it, the first available one is assigned. If a task is assigned it runs a part of it. Then if it is completed, it indicates that no task is assigned. In case of not completing the task, it will be executed in subsequent iterations.

When the master thread finishes the execution of tasks, it enters to the execution of rounds. The first step consists in extraction from the ROSE of the next round to be executed. If a round is able to run, the master node tries to capture it. This action is necessary since there may be outstanding operations with round in the middle of its execution, in which case it could lead to system malfunction. If there is one available round or it has not been captured, it returns to the main loop to inquire if it continues its execution. In case of capturing the round, it runs to completion, after which it returns to the beginning of the main loop.

### 3.2. Server Task

In order to access remotely to the devices network connected to TTP/A bus, a server module has been implemented, linked to the master node. This server supports remote requests to operate on the master node acting as a door to the outside world. The main features of the server are:

- The use of sockets.
- The implementation of multithreading.
- Easily expandable modular architecture.

The server has been implemented so that its execution is performed through threads. This allows us to maintain this module as independent as possible for its development and maintenance and also to be linked enough to the TTP/A thread to interact with itself when necessary.

**Figure 10.**
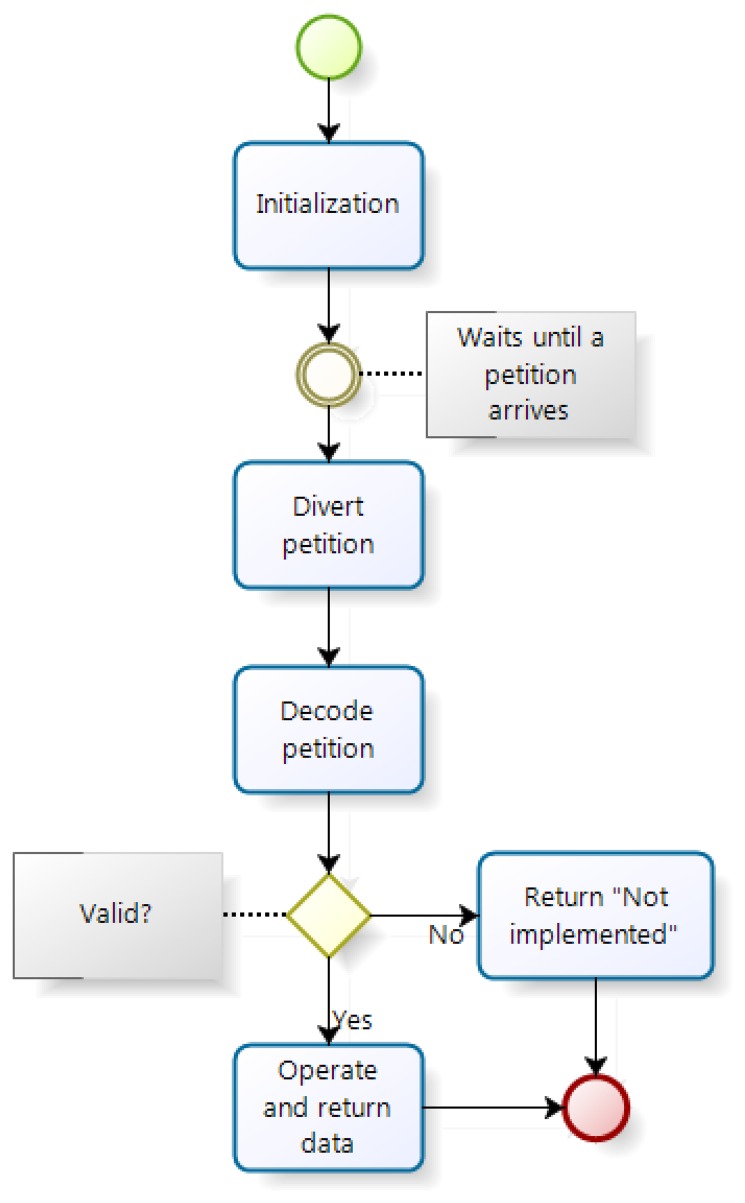
Server threads flow of execution.

The server operation scheme is shown in Figure 10. It is easily seen that the server behavior is similar to a common server. At the beginning of its execution, the server is initialized preparing itself to receive connections. Then, it keeps listening for a request through a socket created in the port that has been assigned to it. Once a request is received is delegated to a new thread to attend the petition while the main thread continues to run the server back to the beginning of the loop waiting for new requests. The new thread created in order to serve the request without blocking the server, decodes the request and acts accordingly; if the operation is not valid the thread reports it by sending the answer to the origin of the request and ends. If it is a valid request, the server generates the response just before the end of its execution processing the necessary data. The priority of all threads derived from server and its main thread is less than the protocol priority that handles TTP/A to keep execution times within its requirements.

Some server operations are responsible for receiving and sending the necessary data for system configurations and others to provide us information about itself. Since some of the operations must be carried out using the TTP/A protocol, both its beginning and its end are conditioned by it. For example, slaves programming cannot be done in real time because it would interfere with the execution rounds. Thus, the programming is stored in an intermediate task buffer from which information is extracted to send interlaced MSA/MSD rounds until its completion.

### 3.3. Web Interface Design

Finally, and to have a functional access to the master, a web interface has been designed to be executed on a dynamic page web server; in our case we used the Boa web server, a lightweight web server with support for dynamic pages execution through CGI.

**Figure 11.**
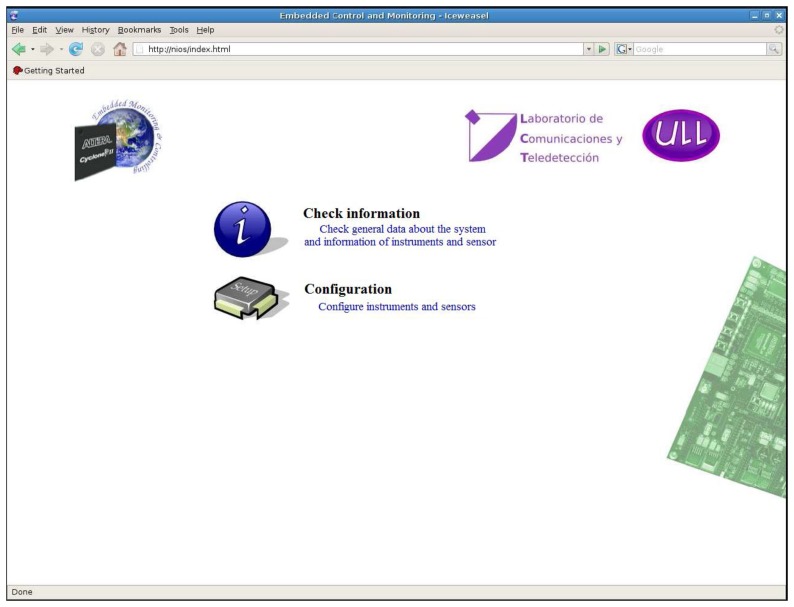
Web interface homepage.

The appearance of the interface is shown in Figure 11. The interface is divided into two different parts differentiated according to its functionality: to query information and to make the configuration. In the first section the operations involve only reading values from the system, *i.e.*, display data of interest. In the second one, we conduct the adjustment of the operations parameters, *i.e.*, all operations that change the system behavior.

While the current dynamic page technology has advanced greatly, in our implementation we could not use any modern technologies such as PHP, Java or .NET to implement the webserver due to its lack of support for the development platform. Even if it would be available, its use of resources to execute the server on the development board would be excessive. Thus, one possibility explored for development was the use of shell scripts, but it was not successful because the shell only offered basic features and the web server support was very limited. This interface became developed through a mixture of static HTML pages and small programs in C language acting as clients of the master node, showing a part of the graphical user interface based on CGI technology. The disadvantage of this solution is that it increases development time. On the other hand, the advantage is the execution code is improved in time and memory usage. This makes it a valid alternative for embedded systems [18].

## 4. Results and Discussion

The whole system was implemented in a Cyclone II EP2C35 FPGA that is in the NIOS II development board Embedded Development Kit. This FPGA is configured on a standard NIOS II processor with μClinux as the operating system. On this system we have implemented an embedded web server and the master node with tasks related to TTP/A protocol and services with the outer world. The prototype employs 2065 LES of the FPGA hardware resources (about 7%), including the core processor and all peripherals used in the design.

In order to debug and verify the operation of the master node a virtual environment was developed to simulate a sensor network where slave nodes had no particular function, *i.e.*, the virtual slaves (software) only execute the operations concerning the TTP/A protocol according on the content of their RODL. This allows debugging to be done on an available PC with two RS-232 ports, connecting a crossover cable between the ports, and executing master and slave nodes on different ports.

It should be noted that the virtual slave device, to be simpler, was first debugged in a separately environment, simulating the serial communications through files, a method which was adopted later to perform different tests of the master node.

Once launched operation for both programs can be checked through the output provided by the system console, and according to the operations indicated for its execution through the web interface. Each operation was executed with different parameters checking at each moment the processes used and outcomes.

Debugging the web interface was done using an apache web server by printing the associated error messages to a *log* file. Once the required performance level was reached, and as the different tasks were implemented, development was moved to the board, creating the image to upload and execute on-board, returning to verify if the execution was correct. The on-board implementation was functional, serving interface pages and correctly generating results from both the interface and the master node.

## 5. Conclusions and Future Work

We have implemented a web interface that can be used as an affordable and versatile control system, applicable to the control of a large number of sensor nodes or instruments, either remotely or within a private network or via Internet. The design has been done using a Cyclone II FPGA to support hardware, configured with NIOS II embedded microprocessor which supported the implementation of an embedded web server. The use of these technologies has yielded a flexible prototype with a relatively short development time.

The implementation of the system control application has been made in C language reducing its complexity. In addition, other master controls can be added to control more devices if they have the necessary communication routes to control the new slaves.

The chosen interface effectively accomplishes its objectives while its use is easy contributing to an acceptance of the system by its users.

By defining the TTP/A protocol as well as the architecture of the implemented system, which allows independence of the user interface, the design is open to future improvements of the protocol as well as other related to the interface above, applying new technologies that maybe developed in the future.

As future improvements to the system are the following ones:

- Identification of the type of connected device, through the use of certain areas of the IFS (Interface File System) node.
- Development of control instrument templates. Once the type of device is identified it could be associated with a given functionality and display a consistent programming interface.
- Integration of TTP/A master as a NIOS II hardware peripheral embedded in the FPGA, rather than the software implementation. This peripheral will be presented to the system through a control implemented in the kernel that allows the sending of tasks to itself and its execution at an indicated time. In this way we will increase the communication speed and keep the developed server. The VHDL implementation of the controller has already been undertaken [9].
- Development of the global system over a FPGA with an embedded hardware processor like ZYNC [19]
